# Social housing of non-rodents during cardiovascular recordings in safety pharmacology and toxicology studies

**DOI:** 10.1016/j.vascn.2016.03.004

**Published:** 2016

**Authors:** Helen Prior, Anna Bottomley, Pascal Champéroux, Jason Cordes, Eric Delpy, Noel Dybdal, Nick Edmunds, Mike Engwall, Mike Foley, Michael Hoffmann, Robert Kaiser, Ken Meecham, Stéphane Milano, Aileen Milne, Rick Nelson, Brian Roche, Jean-Pierre Valentin, Gemma Ward, Kathryn Chapman

**Affiliations:** a National Centre for the Replacement, Refinement & Reduction of Animals in Research (NC3Rs), UK; b Centre de Recherches Biologiques (CERB), France; c Pfizer, USA; d Biotrial, France; e Genentech, USA; f AstraZeneca, UK; g Amgen, USA; h Covance, USA; i Bayer, Germany; j Charles River Laboratories, USA; k Envigo, UK; l WIL Research, France; m Charles River Laboratories, UK; n Abbvie, USA; o WIL Research, USA; p UCB Pharma, Belgium; q GlaxoSmithKline, UK

**Keywords:** 3Rs, Replacement, Refinement And Reduction, AAALAC, Association for Assessment and Accreditation of Laboratory Animal Care International, ARRIVE, Animal Research: Reporting of In Vivo Experiments, AWERB, Animal Welfare and Ethical Review Body, CCTV, closed circuit television, CRO, contract research organisation, ECG, electrocardiogram, GLP, good laboratory practice, IACUC, Institutional Animal Care and Use Committee, ICH, International Conference on Harmonisation, MTD, maximum tolerated dose, NC3Rs, National Centre for the Replacement, Refinement and Reduction of Animals in Research, NCEs, new chemical entities, NHP, non-human primate, R&D, research and development, SPS, Safety Pharmacology Society, 3Rs, Dog, Methods, Minipig, Non-human primate, Non-rodents, Safety pharmacology, Social housing, Telemetry, Toxicology

## Abstract

**Introduction:**

The Safety Pharmacology Society (SPS) and National Centre for the Replacement, Refinement & Reduction of Animals in Research (NC3Rs) conducted a survey and workshop in 2015 to define current industry practices relating to housing of non-rodents during telemetry recordings in safety pharmacology and toxicology studies. The aim was to share experiences, canvas opinion on the study procedures/designs that could be used and explore the barriers to social housing.

**Methods:**

Thirty-nine sites, either running studies (Sponsors or Contract Research Organisations, CROs) and/or outsourcing work responded to the survey (51% from Europe; 41% from USA).

**Results:**

During safety pharmacology studies, 84, 67 and 100% of respondents socially house dogs, minipigs and non-human primates (NHPs) respectively on non-recording days. However, on recording days 20, 20 and 33% of respondents socially house the animals, respectively. The main barriers for social housing were limitations in the recording equipment used, study design and animal temperament/activity. During toxicology studies, 94, 100 and 100% of respondents socially house dogs, minipigs and NHPs respectively on non-recording days. However, on recording days 31, 25 and 50% of respondents socially house the animals, respectively. The main barriers for social housing were risk of damage to and limitations in the recording equipment used, food consumption recording and temperament/activity of the animals.

**Conclusions:**

Although the majority of the industry does not yet socially house animals during telemetry recordings in safety pharmacology and toxicology studies, there is support to implement this refinement. Continued discussions, sharing of best practice and data from companies already socially housing, combined with technology improvements and investments in infrastructure are required to maintain the forward momentum of this refinement across the industry.

## 1 Introduction

The assessment of cardiovascular function (electrocardiogram (ECG), heart rate and blood pressure) within a non-rodent species is a regulatory requirement for all new chemical entities (NCEs) prior to first administration in man (ICH, 2001 S7A; ICH, 2005 S7B). This is usually performed as a safety pharmacology telemetry study (Leishman, Beck, Dybdal, Gallacher, Guth, Holbrook, Roche, & Wallis, 2012) in species such as the dog (Chaves et al., 2007, Gauvin et al., 2006), Göttingen minipig (Authier et al., 2011, Markert et al., 2009) or cynomolgus macaque (Authier et al., 2007, Chaves et al., 2006). With the development of non-invasive jacketed telemetry for dogs (Chui et al., 2009, Prior et al., 2009, Ward et al., 2012, Kremer et al., 2015), minipigs (Ascah et al., 2015, Diehl et al., 2015) and cynomolgus monkeys (McMahon et al., 2010, Derakhchan et al., 2014), cardiovascular endpoints may be integrated into toxicology studies (Authier et al., 2013, Redfern et al., 2013, ICH, 2009a M3(R2)), providing valuable data following repeat-dosing. For biopharmaceuticals or anti-cancer agents the toxicology studies may be the only source of cardiovascular data, as a stand-alone safety pharmacology study is not always required (ICH, 2011 S6(R1); ICH, 2009b S9; Vargas, Bass, Breidenbach, Feldman, Gintant, Harmer, Heath, Hoffmann, Lagrutta, Leishman, McMahon, Mittelstadt, Polonchuk, Pugsley, Salata, & Valentin, 2008).

Housing practices that support good welfare are a top priority in designing a scientifically sound study to obtain high quality cardiovascular data. To generate more representative baseline physiology, avoid abnormal behaviour and for psychological wellbeing, social species, including many commonly used laboratory mammals, should be housed in stable, compatible pairs or groups (social housing). Dogs, minipigs and NHPs exist in groups in nature and social interaction is known to be required for psychological well-being (Prescott et al., 2004, Ellegaard et al., 2010, Jennings and Prescott, 2009). When housed in social groups within the laboratory environment, these animals are able to cope more effectively with potential stressors, exhibit less stereotypical behaviour and have a more balanced temperament (see DiVincenti & Wyatt, 2001 for a review). Additionally, pair-housed dogs have been shown to have lower heart rates and fewer vocalisations (Klumpp, Trautmann, Markert, & Guth, 2006), or fewer stereotypic behaviours (Hetts, Clark, Calpin, Arnold, & Mateo, 1992) than dogs housed alone, whilst isolated pigs show a chronic stress response when compared to group housed pigs (Kanitz et al., 2004, Ruis et al., 2001a). Accordingly, the regulations and policies surrounding housing of non-rodents specially mention the requirement for social housing (e.g., 2010/63/EU, 2010, NC3Rs, 2006, Institute for Laboratory Animal Research, 2011). These are often supplemented by non-governmental systems of oversight, such as voluntary participation by institutions in accreditation programmes (e.g., AAALAC international), company global animal welfare processes (e.g., AWERB/IACUC), and the peer review processes and grant terms and conditions of research funding bodies (e.g., ARRIVE guidelines; Kilkenny et al., 2010, McGrath et al., 2010). The most current updates to international animal welfare regulations specifically state ‘except those which are naturally solitary, shall be socially housed in stable groups of compatible individuals' (Directive 2010/63/EU, 2010) and ‘single housing of social species should be the exception’ (Institute for Laboratory Animal Research, 2011).

Although it is a general practice to socially house non-rodents on safety pharmacology and toxicology studies on non-telemetry recording days, the majority of the pharmaceutical industry will separate animals for data collection on the specific telemetry recording days within a study (Ewart et al., 2013, McMahon et al., 2010, Ward et al., 2012). This separation of animals is considered by some to be justified to allow conduct of the specific scientific procedure (telemetry recording), such that the overall objectives of the experiment can be achieved. Although animals are usually acclimatised to these instances of periodic isolation, this can contribute to increases in heart rate (Xing, Lu, Hu, Wang, Zhao, Zheng, Schofield, Oldman, Adkins, Yu, Platz, Ren, & Skinner, 2015) which may impact study data. However, with the goal of social housing as default, the industry has come together to investigate ways to move towards these preferred conditions. Integration of cardiovascular recordings into repeat-dose toxicology studies, and/or the requirement for longer duration or more frequent recording periods within a study (e.g., for compounds with long pharmacokinetic half-lives, or for biologicals with unique mechanisms of action), may lead to increasing amounts of isolation time compared to ‘standard’ safety pharmacology studies. This further emphasises the importance of refinement of housing practices during telemetry recordings.

Historically, single housing of animals has partly been a consequence of limitations in the equipment used (e.g. spatial separation of animals was required to avoid cross-talk between signals transmitting on the same frequency). However, some systems have been available that allowed and supported recording from multiple animals within the same area due to transmissions on multiple frequencies (Klumpp et al., 2006, Markert et al., 2012, Ward, 2009) but it has not been a standard feature across telemetry manufacturers. Recent technological advances now offer multiple frequencies to be transmitted and recorded as standard, thus increasing the feasibility of wider adoption of recordings from socially housed animals (Bétat et al., 2014, Kearney et al., 2016 (this issue); Milano et al., 2013, Niehoff et al., 2014, Tichenor et al., 2016 (this issue)).

Although this technological advance would address one of the major barriers to implementation of social housing during telemetry recordings, many companies still require additional information before investing in new equipment and infrastructure. One key barrier to change is that the validation of telemetry data acquisitions systems and generation of subsequent historical data sets have been mainly from single housed animals. There is also a perception that recordings from socially housed animals may be more variable than (or otherwise distinct from) single-housed animals due to increased interactions with pen/cagemates, leading to a decreased sensitivity (statistical power) to detect changes in cardiovascular parameters. Nevertheless, some companies have started to validate processes and approaches for running safety pharmacology and toxicology studies using animals grouped together in pairs, threes or fours for the duration of the study (Kaiser et al., 2015, Prior et al., 2015, Xing et al., 2015), in addition to those companies for whom this has been the standard for many years (Klumpp, Trautmann, Markert, & Guth, 2006).

In 2015 the SPS (http://www.safetypharmacology.org) and the NC3Rs (http://www.nc3rs.org.uk) established a working group to collect information on current housing practices of non-rodents during the collection of cardiovascular telemetry data on both safety pharmacology and toxicology studies. Further objectives were to share experiences and canvas opinion on the study procedures/designs that could be used for socially housing animals during telemetry recording periods and what barriers exist preventing this. The working group is facilitated by the NC3Rs and currently represents seven global pharmaceutical companies and six CROs (see author affiliations). The working group contains some companies with experience of successfully implementing social housing to others with no experience of this. The data within this manuscript represent the outcome of an international industry survey conducted in the summer of 2015 and a workshop at the SPS Annual Meeting in October 2015.

## 2 Methods

The questionnaires were sent to targeted individuals within the safety pharmacology and toxicology communities worldwide, with responsibility for either running studies in-house or for outsourcing this work. Different questionnaires were sent to CROs and Sponsors, to allow for the capture of information from groups who run studies themselves (categorised as CRO or Sponsor in-house) and those that outsource studies (Sponsor outsource).

### 2.1 Survey questions

Questions were asked around the themes below, for safety pharmacology and toxicology studies separately, and with separate responses for dog, minipig and NHP (specified as the cynomolgus macaque) as appropriate:

(1) How do you house non-rodents on telemetry recording and non-recording days?
(2) What are the barriers preventing the social housing of non-rodents on recording days? (respondents were requested to choose multiple reasons from a list and then indicate the major reason)
(3) What are the barriers preventing the utilisation of a companion animal on recording days in safety pharmacology studies?
(4) Do you have any positive or negative experiences with social housing during recording sessions?
(5) Is there an impact on the data quality if the animals are socially housed during telemetry recording?
(6) How many animals, and which sex, do you use for safety pharmacology telemetry studies?

Comment boxes were provided throughout the survey to allow submission of additional information and expression of any concerns. Definitions were clarified within the survey as follows:

(1) Social housing: an animal is housed with at least one pen/cage mate for 24 h a day (apart from veterinary reasons for single housing);
(2) Partial social housing: an animal is housed with at least one pen/cage mate for the majority of the day, apart from during study procedures (e.g., separation for recording of food consumption or clinical sign observations immediately post-dose);
(3) Individual housing: an animal is housed alone in the pen/cage (note, the animal may or may not have contact with other animals through the bars or adjoining pens/cages).
(4) Companion animal: an additional animal is housed in the same pen/cage as the recorded animal. This animal may be part of the study (e.g., dosed and recorded on a different day) or may be an undosed animal (e.g., part of the stock colony) provided solely for companionship.

Respondents were requested to provide answers focussed on the most representative/most frequently run cardiovascular telemetry study designs at their facility. Therefore, the responses may relate to early (non-GLP) investigative studies and/or ‘regulatory’ GLP studies intended for regulatory submission. Respondents were requested to provide answers for routine regulatory toxicology studies.

## 3 Results

Multiple replies were received from some companies, reflecting the presence of these companies at different sites worldwide. As these sites may have different standard practices, they were included in the analysis as separate replies. Where a Sponsor only outsourced studies, their opinions regarding the barriers to the adoption of social housing were included in the results.

**Fig. 1:**
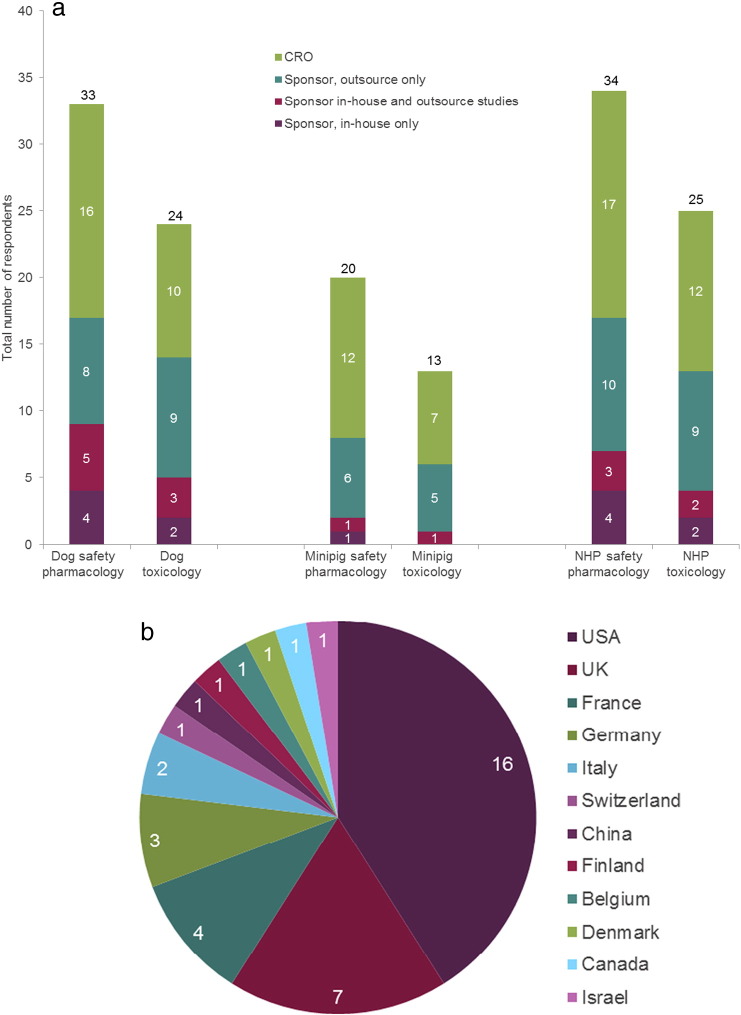
a: Survey respondents per study type and species. The number of respondents to the survey from Sponsors (running in-house studies and/or outsourcing studies) and CROs, for safety pharmacology and toxicology studies in the different non-rodent species. The numbers within the bars gives the actual number of respondents, for ease of reading. b: Survey respondents by geographical location. The overall number of respondents to the survey by geographical location. The numbers within the chart gives the actual number of respondents, for ease of reading.

Eighteen CRO and 21 Sponsor sites responded. 72% ran safety pharmacology telemetry studies and 44% ran toxicology studies with cardiovascular telemetry recording in their facility in at least one of the species. The number of replies across the different species and types of studies are presented in Fig. 1a. A larger number of Sponsors conduct safety pharmacology studies in-house when compared to toxicology studies, which the majority of Sponsors appear to outsource. Replies were received mainly from companies within USA (41%) or Europe (51%), (Fig. 1b).

### 3.1 Safety pharmacology responses

#### 3.1.1 How many animals are used on telemetry studies

**Fig. 2:**
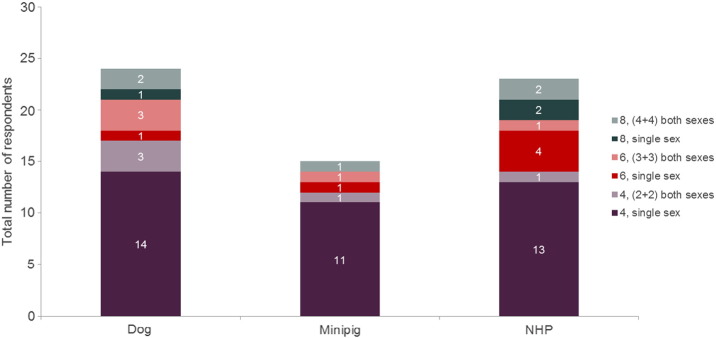
Number and sex of animals used on safety pharmacology telemetry studies. The number of respondents using between 4 and 8 animals, and a combination of single or both sexes, on safety pharmacology telemetry studies. The numbers within the bars gives the actual number of respondents, for ease of reading.

The majority of respondents used four animals of a single sex for safety pharmacology studies. However, there was some variation in the number of animals used and evaluation of both sexes (Fig. 2), increasing the number up to eight animals per study. There was little difference in the number of animals used according to species, with the majority of respondents using the same number of animals regardless of species (i.e., 4, 6 or 8).

#### 3.1.2 Acclimatisation and housing on non-recording and recording days

Acclimatisation procedures vary widely across the respondents, according to where in the facility animals are housed in relation to stock colonies. 54% of responses indicated that animals are housed in the same room as the recording pen/cages, but might move to another pen within the room for the recording period. Some animals are housed in a different room outside of studies, but move to recording pens/cages in a dedicated room for studies. For these animals, acclimatisation times to the recording pen/cage range from 0 to > 12 days, however most respondents (75%) acclimatise for 1–6 days. The majority of respondents (79%) indicated that the animals then remain in the recording room throughout the study rather than being moved back to a stock area between recording sessions.

**Fig. 3:**
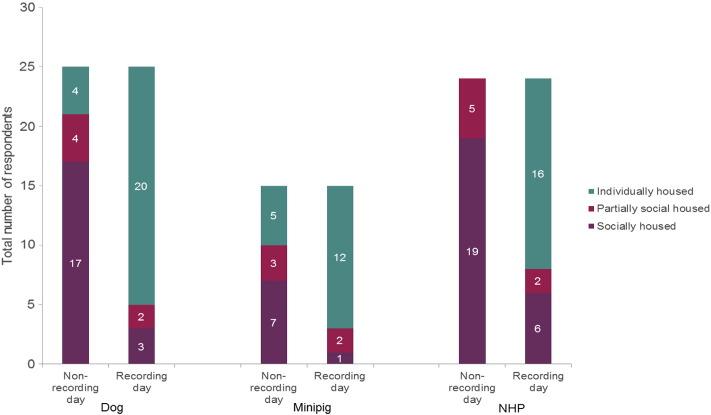
Housing on recording and non-recording days (safety pharmacology studies). The number of respondents housing their animals socially or individually, on recording or non-recording days within a safety pharmacology telemetry study. The numbers within the bars gives the actual number of respondents, for ease of reading.

The majority of respondents social house or partially-social house their animals on non-recording days (84, 67 and 100% of respondents for dogs, minipigs and NHPs respectively). However, this reduces on recording days to 20, 20 and 33% of respondents, respectively (Fig. 3). The majority (7/9) of respondents that house socially during the recording period are from European countries and this practice is slightly more prevalent in studies using NHPs than dogs (5 vs 8).

#### 3.1.3 Barriers for social housing during safety pharmacology telemetry studies

Reasons for not housing animals socially during recordings were similar across species (Table 1, Safety Pharmacology Telemetry column). The top three major reasons were i) limitations of the recording equipment, ii) study design requirements and iii) temperament/activity of the animals. The majority of respondents listed four to six reasons preventing social housing, indicating that although the limitations in recording equipment is the major barrier, there are still many other barriers.

#### 3.1.4 Companion animal use

If the equipment does not allow recording of telemetric data from multiple animals in the same area, one option to maintain social housing is to use a companion animal. Some respondents had experience implementing this approach (7/25 for dog, 3/15 for minipig and 6/23 for NHP respondents).

Reasons for not housing animals with a companion were similar across the species and included the risk of cross-contamination, sponsor expectations, fate of the un-dosed animal, impact on costs/size of colony and size of cages/number of available cages (Table 1, Companion Animal Telemetry column). The majority of respondents listed one to three reasons preventing social housing with a companion, indicating that although the risk of cross contamination to the un-dosed animal is the major barrier, there are still other reasons and barriers to overcome.

#### 3.1.5 Data quality responses

The majority of respondents with experience of social housing for safety pharmacology studies indicated that the data quality was the same or better than data obtained from individually housed animals (9/10 for dog, 4/4 for minipig and 10/11 for NHP respondents, respectively). Further expansion of these qualitative answers was not requested within the survey.

#### 3.1.6 Plans for changing to social housing

**Fig. 4:**
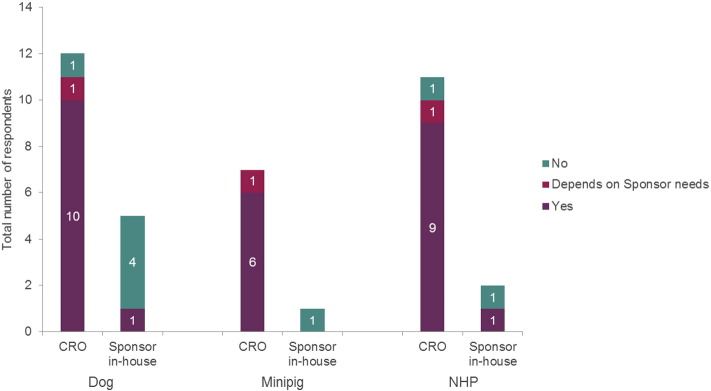
Plans for implementation to social housing during recordings (safety pharmacology studies). The number of respondents planning to implement social housing of animals during recordings in safety pharmacology telemetry studies. The numbers within the bars gives the actual number of respondents, for ease of reading.

Overall, the industry is receptive to social housing during recordings on safety pharmacology telemetry studies and some companies have already implemented social housing (CROs: 3 dog, 2 minipig and 5 NHP respondents; Sponsors: 2 dog, 1 minipig and 3 NHP respondents). Other companies are considering social housing in the future (Fig. 4); however, a different pattern is evident between CROs and Sponsors running studies in-house. For CROs, 10/12 dog respondents, 6/7 minipig respondents and 9/11 NHP respondents who had not yet implemented social housing were actively planning to do so. Conversely for Sponsors, only 1/5 dog respondents, 0/1 minipig respondents and 1/2 NHP respondents who had not yet implemented social housing were actively planning to implement this.

### 3.2 Toxicology responses

#### 3.2.1 Methods currently used to measure cardiovascular data within toxicology studies

There are a number of possible methods utilised for the recording of cardiovascular data within regulatory toxicology studies:

(1) snapshot approach (use of external ECG electrodes and blood pressure cuff attached for short periods of time at specific timepoints and days within the study),
(2) use of a jacketed telemetry system (external ECG electrodes attached, sometimes with blood pressure cuff or minimally-invasive blood pressure implants in addition, covered with a jacket holding the hardware for continuous telemetric transmission on specific days within a study), and
(3) implanted telemetry device (surgical implantation of telemetry hardware for continuous telemetric transmission of ECG and blood pressure waveforms on specific days within a study).

The majority of respondents have the capability to utilise all three of these methods within their facilities (Table 2). However, the percentage of studies performed with jacketed telemetry technology was low, with most respondents (65%) indicating that only 1–40% of their studies used jacketed telemetry (Table 3). For the remainder of the questions the focus was only on studies using jacketed telemetry, as this method of recording may impact the housing of the animals. [Snapshot ECG collections (the second most commonly used toxicology method) has no impact to housing practices, as animals are removed to procedure rooms for this. Implanted telemetry technology will encounter the same barriers as the safety pharmacology recordings described previously.]

#### 3.2.2 Acclimatisation to jackets

Unlike safety pharmacology studies, the majority of respondents indicated that the animals remain in the same pen/cage for the recording sessions as for the rest of the toxicology study (e.g. animals are either individually housed throughout the study, or the pens for socially housed animals are divided and animals are kept separate for recordings). The majority (75%) of respondents acclimatise the animals to the jackets prior to the first recording occasion only. The number of acclimatisation sessions ranged from 0 to 9, with the majority of respondents indicating that they acclimatised for 1–3 occasions prior to the first recording session. There were no apparent differences in the number of acclimatisation sessions used for the different species.

#### 3.2.3 Housing on non-recording and recording days

**Fig. 5:**
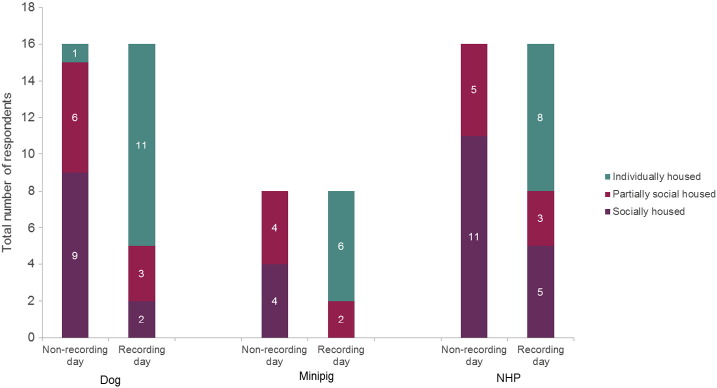
Housing on recording and non-recording days (Toxicology studies). The number of respondents housing their animals socially or individually, on recording or non-recording days within a toxicology study. The numbers within the bars gives the actual number of respondents, for ease of reading.

The majority of respondents social or partially-social house their animals on non-recording days (94, 100 and 100% of respondents for dogs, minipigs and NHPs respectively). However, this reduces on recording days to 31, 25 and 50% of respondents, respectively (Fig. 5).

For respondents that house socially during the recording period there was a similar number of European (5) and non-European (4) countries, however, similar to the safety pharmacology results this practice was more prevalent in studies using NHPs than dogs.

#### 3.2.4 Barriers for social housing during toxicology studies

Reasons for not housing animals socially during recordings were similar across the species (Table 1, Toxicology Telemetry column). The top four major reasons were i) potential damage to the recording equipment, ii) technical limitations of the recording equipment, iii) food consumption recording and iv) temperament/activity of the animals. The majority of respondents had several reasons for not housing socially, with some citing up to eight reasons, indicating that although the potential damage to equipment is the major barrier, there are still many other barriers.

#### 3.2.5 Data quality responses

The majority of respondents with experience of social housing during toxicology studies indicated that the data quality was the same or better than from individually housed animals (8/9 for dog, 3/3 for minipig and 8/9 for NHP respondents, respectively). Further expansion of these qualitative answers was not requested within the survey.

#### 3.2.6 Plans for changing to social housing

Although a low number of responses were received for this question, some companies are considering social housing during cardiovascular recordings within toxicology studies in the future. For CROs, 2/4 dog respondents, 1/1 minipig respondents and 2/3 NHP respondents who had not yet implemented social housing were actively planning to do so. For Sponsors, 1/3 dog respondents and 0/1 NHP respondents who had not yet implemented social housing were actively planning to implement this. A similar number of CROs and Sponsors have already implemented social housing (CRO: 4 dog, 2 minipig and 5 NHP respondents; Sponsor: 1 dog, 0 minipig and 3 NHP respondents).

## 4 Discussion

The purpose of this survey and the subsequent workshop was to describe current practices for housing non-rodents during cardiovascular telemetry recordings in safety pharmacology and toxicology studies, with the further goal of identifying opportunities for the expansion of social housing across entire study durations. Although the majority of respondents already house animals socially on non-recording days, most revert to individual housing on days when cardiovascular data is acquired. However, many respondents were in favour of implementing social housing during recordings, with some companies already evaluating conducting studies this way, and others considering this change. A number of concerns were raised within the survey, which will require the drug development industry as a whole to address in order to increase acceptance and adoption of this refinement. Whilst most of the respondents were based within Europe and the USA, we received an appropriate number of responses from the facilities actively running these studies (within both CROs and pharmaceutical companies), and from the companies outsourcing this work to CROs to have a representative view of the industry at-large.

With the recent decline in R&D productivity (increases in R&D costs with output of new drugs remaining static, Cook, Brown, Alexander, March, Morgan, Satterthwaite, & Pangalos, 2014) better and more efficient strategies are urgently required. However, changes to established ways of working can require careful consideration and validation. Ideally, a change in drug development processes would result in a reduction in costs and timelines, whilst improving data quality. In reality, although it is not always possible to improve all three, it is still advantageous to improve one whilst maintaining the others. The challenge for the industry is to identify and apply the benefits of social housing in terms of animal welfare, quality of science, cost and drug development timelines.

As seen in Table 1, there is a wide range of reasons cited for not implementing social housing during telemetry recordings. These reasons can be generally divided into ‘physical aspects’ (e.g. the size of pens/cages, or the telemetry equipment available within the facility) or ‘risk perceptions’ (e.g. the expectation of reduced data quality or the risk of cross-contamination). Sharing of experiences and demonstration of the successful use of socially housed animals in these studies may help overcome some of the assumed risks. Information within this manuscript is intended to provide reassurance and potential solutions to those facilities that are planning to use socially housed animals in the future. For facilities that have not yet considered social housing, information within this manuscript may lead to some discussions for interim measures and/or future refinements.

This survey has highlighted an increase in outsourcing of studies encompassing cardiovascular measurements compared with previous surveys (Ewart et al., 2012, Lindgren et al., 2008), as organisational changes within the pharmaceutical industry continue the trend for consolidating work at CROs (Ewart, Milne, Adkins, Benjamin, Bialecki, Chen, Ericsson, Gardner, Grant, Lengel, Lindgren, Lowing, Marks, Moors, Oldman, Pietras, Prior, Punton, Redfern, Salmond, Skinner, Some, Stanton, Swedberg, Finch, & Valentin, 2013). The survey also shows a preference of pharmaceutical companies to use socially housed animals in studies involving telemetry recording. However, the lack of capital investment to support these changes in-house may be partly responsible for the expansion in outsourcing. The survey indicated that very few pharmaceutical companies run all their safety pharmacology studies in-house, with even fewer running toxicology studies in-house. Most rely either entirely on CROs to run these studies, or in combination with in-house work. Therefore, the increased implementation of social housing may lie primarily within the CRO sector. However, the views and acceptance of Sponsors (mainly within the pharmaceutical and biologicals industry) will also be an important factor, as their requirements will influence the CRO market. The survey indicated a need to improve communication on this topic as Sponsors commented that they would appreciate the availability of social housing options at CROs, whereas comments from CROs indicate there are few requests for these conditions from Sponsors.

The main barriers to adoption of social housing are discussed below and are summarised in Table 4, Table 5, Table 6.

### 4.1 Limitations in recording equipment

Social housing is facilitated when using a telemetry system having independent frequencies for each animal. Although the recent introduction of new technologies should enable the concept and methods for social housing during telemetry recordings to be available to a wider number of companies, the principles of this have been available to users of other technologies for some time. Whilst some groups using these alternative technologies have socially housed during safety pharmacology (Klumpp et al., 2006, Markert et al., 2012) or toxicology studies (Ward, 2009), others did not (Prior et al., 2009, Chaves et al., 2007, Sivarajah et al., 2010). This difference is likely to be due to many of the reasons highlighted within this survey. Many of the companies surveyed will upgrade to new equipment in the future that allows for social housing, as the sale of and/or support for older systems are phased out by the manufacturers. Therefore, the most common technical barrier to the adoption of social housing during recordings will disappear over time.

### 4.2 Pen/cage size and room layout

Individual facilities are unique in the way their telemetry recording rooms and stock colony holding rooms are arranged, and it can be difficult to make major changes to this infrastructure (requiring time and capital investment) unless there is a specific reason or desire to do so. European facilities comply with, or exceed, the updated animal welfare laws (Directive 2010/63/EU, 2010) so these pens tend to have larger available areas per animal, which may facilitate social housing during recordings. Many of these companies have other facilities worldwide (or exchange studies within different countries, so may harmonise practices), and this may increase the uptake of social housing through staff exchanges, technical talks, in-house 3Rs awards, etc. The business/financial decisions to make these infrastructure changes would be strengthened by 1) a change in regional directives (e.g. as observed in Europe), 2) scientific justification/acceptance within the industry and 3) Sponsor requests to CROs.

Some facilities hold animals between studies in large groups in separate rooms, then move animals to individual pens/cages for recording. If these pens/cages have doors/hatches to open up to allow at least two animals to socialise during periods of non-recording, with suitable equipment it should also be possible to allow this to continue during recordings.

### 4.3 Use of a companion animal

If facilities have pens/cages of sufficient size to house two animals (complying with local laws) but their telemetry equipment is not able to record from both simultaneously, the inclusion of an unrecorded companion animal should be considered. One option is for the companion animal to also be part of the study, but dosed and recorded on a different day. However, this could prolong the study duration due to the staggered dosing. Alternatively, the companion animal may be an undosed animal and not included in the study (e.g. part of the stock colony), but instead provided solely for companionship. However, there is a potential risk of cross-contamination of the companion animal, which could restrict possible future use of this animal on another study. There are also concerns that inclusion of a companion animal could increase the overall number of animals used and potentially the size of stock colonies, however, as the companion animal is not undergoing study procedures (dosing, etc), this is still considered a refinement for the study animal. Study designs and concerns around abnormal/increased activity due to the second animal are addressed below.

### 4.4 Changes in animal behaviour/activity and sensitivity of cardiovascular data

There is a growing pool of data available comparing cardiovascular data from socially housed animals with data from individually housed animals, in both safety pharmacology and toxicology study settings. Studies showing no significant difference in cardiovascular data in socially housed compared with individually housed animals have been published in dogs (Kearney, Appleby, Kieper, & Atterson, 2016 (this issue); Prior et al., 2015, Sadekova et al., 2015, Sadekova et al., 2016 (this issue)) and cynomolgus monkeys (Kaiser et al., 2015, Tichenor et al., 2016 (this issue)). In contrast, other publications indicate that lower heart rates were obtained in socially housed cynomolgus monkeys (Bétat et al., 2014, Kreckler et al., 2015, Xing et al., 2015) and dogs (Klumpp et al., 2006, Ward, 2009) compared to individually housed animals. A change in heart rate for socially housed animals could be interpreted a number of ways. For instance: an increase could indicate higher stress levels, or it could be a consequence of more interactions between the animals and/or more movement in what might be a slightly larger pen/cage than used for individual animals. A decrease in heart rate might indicate lower stress in a social setting, or it might indicate animals have ‘settled’ quicker after disturbances as they have a companion to buffer stress (Sanchez et al., 2015, Ruis et al., 2001b). It will be important for those implementing social housing to understand and interpret the effects of social housing on cardiovascular data in their own facilities.

### 4.5 Study design

The standard study design for safety pharmacology studies is the Latin-square cross-over design (Guth, Chiang, Doyle, Engwall, Guillon, Hoffmann, Koerner, Mittelstadt, Ottinger, Pierson, Pugsley, Rossman, Walisser, & Sarazan, 2015), which is widely accepted as conferring the best statistical power (sensitivity to detect changes in cardiovascular parameters of interest; Aylott et al., 2011, Leishman et al., 2012). Some facilities performing studies with socially housed animals have retained the Latin-square design, whereby each animal within the pen receives a different dose level, with no reports of cross-contamination in over 15 years of using this approach, based on drug exposure confirmation with each study (Guth, 2015, personal communication). Other facilities have adopted a slightly more conservative approach, whereby a ‘partial’ Latin-square is used, such that the two animals within the pen receive the same dose level (Prior, Billing, Wallace, South, Oldman, Moors, Edmunds, & Milne, 2015) and report similar sensitivity to previous Latin-square designs. Alternatively, an ascending dose design, whereby all the animals within the pen/cage receive the same dose level may be an even more conservative option, and this design can exhibit similar statistical power (Ewart et al., 2013, Prior et al., 2015, Guth et al., 2009) to Latin-square designs, although may be confounded by day effects. The adoption of a Latin-square study design may be dependent on the known properties of the compound, the dose levels chosen, and the species. For example, it is known that dogs in the laboratory may ingest vomit and/or faeces from pen/cagemates and cynomolgus monkeys spend substantial time grooming/licking pen/cagemates. If the chosen dose level is not expected to cause emesis (e.g. a well-characterised compound, or a test compound where the maximum tolerated dose (MTD) is known) then the risk of cross-contamination may be considered low. Inclusion of minimal blood sampling for confirmation of drug-exposure may be useful to identify any deviations due to cross-contamination within socially housed animals and publications exploring the incidence of cross-contamination using these study designs would be valuable. As study sensitivity and test article-characteristics are unique to each facility, the study design for socially housed safety pharmacology studies needs to be assessed within the validation studies and on a study-by-study basis.

### 4.6 Food consumption/clinical signs monitoring

The measurement of food consumption from individual animals is not a requirement within safety pharmacology studies, however it is often recorded as a measure of general clinical condition of the animal. Depending on the time of day food is offered, and the timing in relation to any critical periods of cardiovascular monitoring, animals could be separated for a short period of time (e.g. 1 h) to allow these data to be obtained, and some facilities are using this approach (this was the definition for partial social housing within the survey). Other facilities simply provide each animal with a separate food allowance and make careful observations on the consumption.

Collection of food consumption data is often a requirement for toxicology studies; however, as animals are generally socially housed on other days within the study, the cardiovascular recording should not impact these data if the same procedures are followed. Efforts should be made, however, to reduce the disturbance to the animals these procedures may cause on the cardiovascular recording days. Whole group or cage assessments for food consumption are acceptable within toxicology studies, particularly for NHPs. Automated systems may be implemented on these types of studies to measure food consumption from socially housed non-rodents, similar to those already available for rodents (Krohn, Farlov, & Hansen, 2011) or to those used within academia (Wilson, Fisher, Fischer, Lee, Harris, & Bartness, 2008) or more sophisticated systems used in pig production facilities e.g. Young & Lawrence (1994).

Clinical signs can be monitored in safety pharmacology and toxicology studies via closed circuit television cameras (CCTV), but a method of individual identification may be required in socially housed animals. Animals could be marked non-invasively on the flank or the head, or different colour jackets could be worn (e.g., see Rose, de Heer, Korte, van der Harst, Weinbauer, & Spruijt, 2012). Clinical signs are typically monitored on non-recording days from socially housed animals on toxicology studies, thus if the same processes are adopted, then it should also be possible on the telemetry recording days.

### 4.7 Damage to equipment (jacketed telemetry)

Jacketed telemetry has been used within the industry for over a decade (Hunter, Schofield, Gracie, Moors, Philp, Carter, Prior, Valentin, & Hammond, 2004) and has allowed the integration of quality continuous cardiovascular assessments into toxicology studies. However, implementation of jacketed telemetry into socially housed conditions could present some issues. Even single housed animals are able to tamper with their jackets and damage/destroy the ECG leads, leading to loss of data (Chui, Fosdick, Conner, Jiang, Bruenner, & Vargas, 2009) and socially housed animals may increase the incidence of this, due to interactions with equipment of pen/cagemates. However, comments within the survey from companies implementing (or validating) social housing suggest that the animals are less prone to jacket destruction, perhaps as they are sufficiently ‘enriched’ by their companion(s) (Kaiser et al., 2015, Sadekova et al., 2015, Xing et al., 2015). The keys to successful jacketed telemetry are (whether animals are singly or socially housed) appropriate jacket design, size and placement, along with sufficient acclimatisation to the jackets, as these will reduce the risk of damage of the jackets during the study. There are a number of articles with thorough descriptions of acclimatisation regimens (Derakhchan et al., 2014, Prior et al., 2009, APV (Association of Primate Veterinarians), n.d). Some ‘tips’ and solutions for successful recordings include introducing animals to wearing jackets from a young age (e.g., at the breeders facility), wearing an undershirt or bandaging or another jacket below the outer jacket (Ascah et al., 2015, Chui et al., 2009, Ward et al., 2012, Xing et al., 2015) to protect the electrodes, modifying the jackets to hide zips (Xing, Lu, Hu, Wang, Zhao, Zheng, Schofield, Oldman, Adkins, Yu, Platz, Ren, & Skinner, 2015). Additional acclimatisation sessions to jackets before each recording session (particularly important for longer studies where there may be a gap of a number of weeks between recording sessions) could also be used. Additionally, some practices may be transferrable from ambulatory infusion toxicology studies which are frequently run in social housed conditions.

#### 4.7.1 Other 3Rs' opportunities highlighted within the survey results

Although the survey was primarily interested in practices and views on social housing during telemetry recordings, a number of answers highlighted some interesting opportunities for additional animal welfare improvements. These are discussed below.

### 4.8 Number of animals used (Reduction)

Most companies use four animals in their safety pharmacology telemetry studies, usually of the same sex. However, some companies use six or eight animals and a mix of both sexes. The number of animals used should depend on the study sensitivity required for a given study type at each specific facility, however, a geographical trend indicated European companies often use fewer animals. Whilst the use of four animals can deliver acceptable test sensitivity for the majority of the measured parameters, there can be small improvements in test sensitivity when increasing to eight animals (Chiang et al., 2007, Sivarajah et al., 2010, Guth et al., 2009). If social housing leads to less variability within the data, there could be opportunities for facilities to reduce the number of animals used, dependent on study requirements. Reducing the number of animals used will have associated reductions in study costs and timelines (technician time, data processing time, etc.).

### 4.9 Housing practices during acclimatisation/non-recording days (Refinement)

The survey highlighted that in 100% of respondent facilities, NHPs are socially housed as stock or on non-recording days. However, dogs are still housed individually in a small proportion of facilities, even as stock or on non-recording days in safety pharmacology studies or for the entirety of toxicology studies. This may be due to infrastructure reasons or concerns around compatibility/conflict within groups. For guidance on successful social housing, please refer to NC3Rs, 2006 and Prescott, Morton, Anderson, Buckwell, Heath, Hubrecht, Jennings, Robb, Ruane, Swallow, & Thompson, 2004. Some respondents expressed concerns about changing the housing conditions within a study, preferring to maintain the animals in a stable condition at all times rather than switching the animals between social and single housing on recording and non-recording days, respectively. These comments provide support for social housing throughout the study. As most facilities are now social housing NHPs on non-recording days, this should increase the adoption of social housing on recording days where technically feasible. There is also the potential for this practice to extend to the other non-rodent species.

### 4.10 Use of minipigs

An unexpectedly high number of respondents run safety pharmacology and toxicology telemetry studies in the minipig (20 and 13 companies, respectively). When the dog is considered unsuitable as the non-rodent for use, the minipig is often the second species of choice and can potentially be a suitable model for many different toxicology study types (Bode, Clausing, Gervais, Loegsted, Luft, Nogues, & Sims, 2010). The acceptable cardiovascular data from these animals (Authier et al., 2011, Markert et al., 2009) and the stability of pairings means that minipigs can be considered as a suitable non-rodent species for telemetry studies in socially-housed conditions.

### 4.11 Conclusions and recommendations

The implementation of social housing during telemetry recordings in safety pharmacology and toxicology studies is an achievable goal. However, individual companies will have varying challenges according to their current practice and infrastructure, which will be unique to each specific facility. For example, changing to social housing may require review/modification of physical aspects such as pen/cage sizes, room availability and telemetry hardware, and/or process aspects such as acclimatisation of animals to each other and to recording areas, stock colony/pair compositions and study designs. Each facility will need to investigate the optimal conditions to ensure the cardiovascular data obtained from socially housed animals is of similar, or better, quality and sensitivity than from individually housed animals. If Sponsors do not wish to invest in their in-house facilities, then the trends identified in the survey shows increased opportunities to use CROs for socially housed studies. CROs need to meet market expectations but concerns over possible decreases in capacity/number of studies that can be offered when using social housing will need to be addressed. CROs may therefore retain capabilities to run studies according to all possible requests as an interim measure. Further demonstration of cost or time-efficiencies, in addition to an increased data quality, will accelerate the implementation of socially housed studies.

**Fig. 6:**
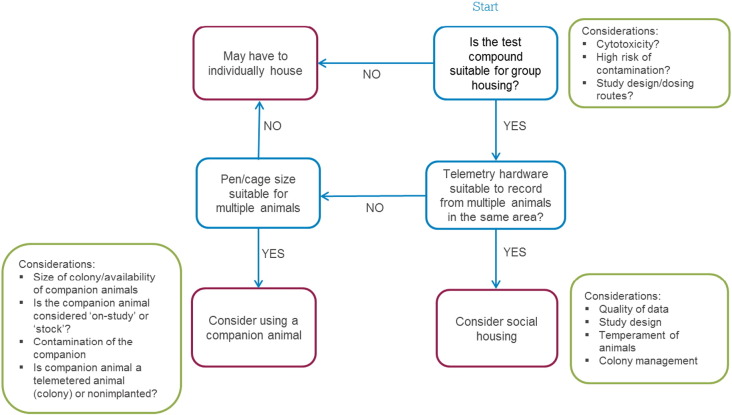
Flow-diagram with decision tree for group-housing on safety pharmacology studies.

The implementation of social housing during telemetry recording may be most straightforward for standard studies initially, e.g. oral dosing studies when using cautious dose levels (to minimise emesis and cross-contamination) if the MTD is already known. There may be specific studies where, in order to ensure the likelihood of successful completion, animals will need to be singly housed (e.g., if using a technical dosing route or a specific compound which may cause aggressiveness or other clinical signs which may affect the welfare of pen/cage mates). However, the overall intention should be to consider social housing as normal/default from the start, and ensure robust scientific rationale is used to deviate from this. Fig. 6 shows a flow diagram with decision points and suggestions for housing options for individual studies. Additionally, pair housing of animals may be more achievable in the short term for some companies than housing and recording from larger groups of animals.

In conclusion, although the majority of the industry does not yet have the experience or capabilities for recording from socially housed animals in safety pharmacology and toxicology studies, there is support to implement this refinement. Continued discussions, sharing of best practice and data from companies already socially housing, combined with technology improvements and investments in infrastructure are required to maintain the forward momentum of this refinement across the industry.

## Conflict of interest statement

There are no known conflict of interests for any authors.

## Figures and Tables

**Table 1:** Reasons stated for not social-housing in safety pharmacology and toxicology telemetry studies.

	Safety pharmacology telemetry^a^			Companion animal telemetry^b^			Toxicology telemetry		
	Dog (29)	Minipig (17)	NHP (26)	Dog (20)	Minipig (11)	NHP (19)	Dog (22)	Minipig (11)	NHP (21)
Limitations of recording equipment currently being used	79	82	92				50	64	52
Study Design	66	76	62						
Damage to equipment				5	0	0	64	55	48
Sponsor requirement/expectation^c^	57 (14)	42 (12)	46 (13)	67 (12)	71 (7)	75 (12)	63 (8)	20 (5)	50 (8)
Housing options available at CRO^c^	60 (10)	50 (4)	36 (11)						
Temperament of individual animals	52	47	50				41	45	38
Monitoring of clinical signs observed	45	24	31				41	27	38
Colony management	21	29	46						
Food consumption recording of individual animals	38	29	27				59	55	48
Size of pen/cage/No. of pens/cages in the recording room	28	35	69	20	27	21	27	36	19
Increased/abnormal activity	34	24	31				32	27	33
Quality of data obtained	34	29	23				36	36	29
Validation of processes required	28	24	23				36	45	38
How the recording room is set-up	24	6	23				23	18	24
Size of animals	3	12	15				9	18	19
Cross-contamination	0	0	4	70	73	68			
Fate of un-dosed animal				45	45	37			
Impact on costs/increase in colony size				20	18	21			
Risks of conflicts between animals				5	0	5			
Availability/compatibility of companion animal				5	9	0			

Data represented is % of respondents (the number in brackets next to species indicates the number of replies received for that species and subset of the survey).

a Where two or more animals have telemetry recordings simultaneously from within the same pen/cage.

b Where only one animal has telemetry recordings but an undosed animal is present within the same pen/cage. For the companion animals' answers, these were different/additional options to the safety pharmacology telemetry answers.

c These questions were only asked of either the CRO or Sponsor and therefore the total number of respondents is different than the other replies and is stated in brackets next to the percentage.

**Table 2:** Methods currently available to measure cardiovascular parameters on toxicology studies.

	Dog			Minipig			NHP		
	CRO (12)	Sponsor (5)	Total (17)	CRO (7)	Sponsor (1)	Total (8)	CRO (13)	Sponsor (4)	Total (17)
Snapshot	11	3	14	6	0	6	12	3	15
Jacketed telemetry	11	5	16	6	1	7	12	4	16
Implanted telemetry	7	2	9	3	0	3	9	1	10

Data presented is number of respondents. Respondents were able to choose multiple options, in brackets is the total number of responses received.

**Table 3:** Percentage of toxicology studies using jacketed telemetry.

	Dog			Minipig			NHP		
	CRO	Sponsor	Total	CRO	Sponsor	Total	CRO	Sponsor	Total
1–40%	7	2	9	3	0	3	8	2	10
41–80%	3	1	4	2	0	2	2	2	4
81–100%	0	1	1	0	1	1	1	0	1

Data presented is number of respondents.

**Table 4:** Potential considerations/recommendations for social housing on safety pharmacology studies.

Major barriers identified in the survey and possible resolutions
Limitations of recording equipment currently being used (e.g. signal cross talk) ▪ Advances in technology allows recording from multiple animals in the same pen/cage ▪ Consider using an unrecorded companion animal if pen/cage size allows Study design (e.g. Latin-square preferred design may lead to cross-contamination risks if different animals receive different dose levels within the same pen/cage) ▪ Consider using a partial Latin square design (e.g. two animals receive the same dose level on each occasion), see Prior, Billing, Wallace, South, Oldman, Moors, Edmunds, & Milne, 2015 ▪ Consider using an ascending dose design ▪ Consider dosing different dose levels within the same pen/cage if test article characteristics suggest low contamination risk ▪ Consider including minimal drug-exposure sampling to monitor incidence of cross-contamination Quality of data obtained ▪ Publications indicate that social housing of animals does not impact the quality of the data in both dogs and NHPs (e.g., Klumpp et al., 2006, Prior et al., 2015) ▪ In this survey, the majority of respondents indicated that the data was the same or better from socially housed animals (8/9 dog, 4/4 minipig and 10/11 NHP respondents) compared to individual housed historical data Colony management (e.g. concerns about introducing a new animal if one has to be removed from the social group) ▪ Ensure close observation and knowledge of individual animals within the colony ▪ Consider removing ‘unruly/disruptive’ animals from the colony ▪ May have to increase the colony size to allow for more combination options once on study Increased/abnormal activity due to the presence of multiple animals in the pen/cage ▪ Ensure sufficient acclimatisation in the study grouping prior to the start of the study ▪ Publications indicate that social housing does not detrimentally affect heart rates in comparison to individual housing (e.g., Kaiser et al., 2015, Klumpp et al., 2006, Prior et al., 2015) Validation of processes required for social housing ▪ Consider performing a validation study in socially housed animals so results can be compared to historical data from individually housed animals ▪ Review statistical power to detect cardiovascular changes in socially housed animals (using various study designs and animal numbers, if appropriate)

**Table 5:** Potential considerations/recommendations for use of companion animal on safety pharmacology studies.

Major barriers identified in the survey and possible resolutions
Cross contamination ▪ Consider the wash out period required for the companion animal (may be dependent on the characteristics of the test item) ▪ Consider the risk of contamination taking in account known information about the test material i.e. dose route, route of excretion, dose levels to be used ▪ Consider taking single proof-of-exposure blood samples Sponsor expectation ▪ Consider providing a data set to Sponsors indicating that the presence of a companion animal has no impact on the data quality Fate of undosed animal after study completion ▪ Consider in-house procedures to ensure that the companion animal can be used as a companion animal on future studies Impact on costs/increase in size of colony ▪ Consider the source of the companion animal, this may be an animal where the telemetry equipment is no longer viable, or an animal that has been rejected from other studies (e.g. from other safety pharmacology or toxicology studies)

**Table 6:** Potential considerations/recommendations for social housing on toxicology studies.

Major barriers identified in the survey and possible resolutions
Damage to the equipment by pen/cage mates ▪ Consider the amount of acclimatisation to the jackets prior to the study start and if further acclimatisation/refreshing should be performed before each recording session during the study ▪ Consider using a second jacket or T-shirt under the outer jacket, to cover the ECG leads ▪ Consider using implanted technology ▪ Kaiser, Tichenor, Regalia, York, & Holzgrefe (2015) indicates that there was no increase in incidence of equipment damage in paired NHPs vs single housed animals Limitation of the recording equipment currently being used (e.g. signal cross-talk) ▪ Consider using alternative technologies which allow for multiple signals to be recorded Quality of data obtained ▪ Publications indicate that social housing does not impact the quality of data in both dogs and NHPs (e.g. Kaiser et al., 2015, Sadekova et al., 2015, Xing et al., 2015) ▪ In companies that had experience of social housing 8/9 dog, 3/3 minipig and 8/9 NHP respondents indicated that the data obtained was the same or better than from individually housed animals Temperament of individual animals ▪ Consider the acclimatisation period prior to the start of study investigations ▪ Hierarchies/dominance ▪ Age of animals being used ▪ Consider working with the supplier to assess compatibility of animals prior to arrival Validation of processes required for social housing ▪ Safety pharmacology studies are publishing data on social housing (Klumpp et al., 2006, Prior et al., 2015) ▪ Consider investigating the implications of social housing during the pre-treatment phase of studies to build up data on processes and impact Monitoring of clinical signs observed in individual animals ▪ Consider the expected effects of test compound (indications from previous work e.g. MTD studies) ▪ Consider the use of CCTV cameras and methods of identifying individual animals (e.g. colour coded jackets) Size of animals ▪ Consider the age/weight of the animals being used ▪ Consider the introduction procedures utilised when the animals first arrive at the site ▪ Consider the acclimatisation to the social groups prior to the start of any investigations
